# Uric acid induced hepatocytes lipid accumulation through regulation of miR-149-5p/FGF21 axis

**DOI:** 10.1186/s12876-020-01189-z

**Published:** 2020-02-18

**Authors:** Shenghui Chen, Dan Chen, Hua Yang, Xinyu Wang, Jinghua Wang, Chengfu Xu

**Affiliations:** Department of Gastroenterology, the First Affiliated Hospital, College of Medicine, Zhejiang University, 79 Qingchun Road, Hangzhou, 310003 Zhejiang Province China

**Keywords:** Non-alcoholic fatty liver disease, Uric acid, miR-149-5p

## Abstract

**Background:**

Hyperuricemia is a major risk for non-alcoholic fatty liver disease. However, the mechanisms for this phenomenon are not fully understood. This study aimed to investigate whether microRNAs mediated the pathogenic effects of uric acid on non-alcoholic fatty liver disease.

**Methods:**

Microarray was used to determine the hepatic miRNA expression profiles of male C57BL/6 mice fed on standard chow diet, high fat diet (HFD), and HFD combined with uric acid-lowering therapy by allopurinol. We validated the expression of the most significant differentially expressed microRNAs and explored its role and downstream target in uric acid-induced hepatocytes lipid accumulation.

**Results:**

Microarray analysis and subsequent validation showed that miR-149-5p was significantly up-regulated in the livers of HFD-fed mice, while the expression was down-regulated by allopurinol therapy. MiR-149-5p expression was also significantly up-regulated in uric acid-stimulated hepatocytes. Over-expression of miR-149-5p significantly aggregated uric acid-induced triglyceride accumulation in hepatocytes, while inhibiting miR-149-5p ameliorated the triglyceride accumulation. Luciferase report assay confirmed that FGF21 is a target gene of miR-149-5p. Silencing FGF21 abolished the ameliorative effects of miR-149-5p inhibitor on uric acid-induced hepatocytes lipid accumulation, while overexpression of FGF21 prevented the lipid accumulation induced by miR-149-5p mimics.

**Conclusions:**

Uric acid significantly up-regulated the expression of miR-149-5p in hepatocytes and induced hepatocytes lipid accumulation via regulation of miR-149-5p/FGF21 axis.

## Background

Nonalcoholic fatty liver disease (NAFLD) is a group of liver disease characterized by excessive hepatic lipid accumulation without excess alcohol intake [1]. It ranges from simple steatosis to steatohepatitis, fibrosis, cirrhosis, and eventually hepatic carcinoma [2]. NAFLD is the most frequent chronic liver disease worldwide, the prevalence of NAFLD in Asia is increasing, and its prevalence in China has rapidly climbed to 29.2% in 2019 [3, 4]. NAFLD is strongly associated with obesity, type 2 diabetes mellitus and cardiovascular diseases, all of which leads to serious public health issues worldwide [5–7]. Despite intensive investigations over past decades, the precise pathogenesis of NAFLD remains poorly understood.

Uric acid is the final enzymatic product of purine metabolism. We previously identified that high serum uric acid level is a major risk factor of NAFLD [8, 9], and uric acid induced hepatic lipid accumulation by activating NLRP3 inflammasome [10]. Uric acid may also induce hepatic lipid accumulation by inducing endoplasmic reticulum stress and mitochondrial oxidative stress [11, 12]. Although increasing researches have emerged to explore the mechanism by which uric acid induced hepatic lipid accumulation, its underlying molecular mechanisms remains not fully clarified. A better understanding of the mechanisms may help for developing novel therapeutic strategy for NAFLD.

MicroRNAs (miRNAs) are members of small non-coding RNAs, which are consist of approximately 18–24 nucleotides. MiRNAs are major in negatively regulate gene expression at the post-transcriptional level by binding to target mRNA followed by silencing or promoting of the mRNA transcription [13]. There are more than 2600 miRNAs have been reported in miRbase and each miRNA can regulate hundreds of gene transcripts [14]. A growing number of studies implicated that miRNAs are participated in the pathogenesis of NAFLD [15]. For instance, miR-122, which is the most abundant miRNA in human liver, was decreased in liver of high fat diet (HFD) fed mice, and miR-122 could prevent hepatic steatosis by decreasing mRNA expression levels of lipogenic genes [16]. Our previous miRNA microarray employed in livers of ob/ob mice and STZ-induced diabetic mice also indicated that many miRNA participate in the pathophysiological processes of NAFLD with hyperglycemia [17]. In addition, another previous study found that miR-34a was elevated in the livers of HFD-fed mice, and miR-34a induced hepatic lipid accumulation through regulating its target gene PPARα [18]. Interestingly, a previous study has reported that a high concentration of uric acid significantly down-regulated miR-92a, which contributed to angiogenesis [19]. However, whether the effects of uric acid on hepatocytes lipid accumulation is mediated by miRNAs remains unclear.

Allopurinol, the prototypical xanthine oxidase (XO) inhibitor, has been considered as the cornerstone of the clinical management of gout or hyperuricemia for several decades [20]. In the previous study, we also have found that lowering the serum uric acid levels with allopurinol significantly alleviated HFD-induced hepatic steatosis in mice [10]. In this study, we aimed to explore the hepatic miRNA expression profiles of HFD-fed mice treated with or without allopurinol-therapy. We found that miR-149-5p is involved in the effects of uric acid on hepatocytes lipid accumulation via regulation of its target gene FGF21.

## Methods

### Animals and treatments

Male C57BL/6 mice, 8–10 weeks of age, weighing 18–20 g, were purchased from the Medical Science Institution of Zhejiang Province (Hangzhou, China). Before experiment, mice were allowed to acclimate for 1–2 weeks access to food and water. The mice were randomly divided into 3 groups, including of 6 animals per group and fed with standard chow diet (SCD), HFD, or HFD combined with allopurinol (120 mg/L) in the drinking water (HFD + A) for 8 weeks. The HFD (D12492, Research Diets, New Brunswick, NJ) contains 60% of kcal from fat, 20% from carbohydrates and 20% from protein. Mice were maintained in a 12-h light/dark cycle at controlled room temperature and given free access to food and water.

After intraperitoneal injection of 5% chloral hydrate, mice were sacrificed by cervical dislocation. The liver of each mice was removed and weighted. After rinsing with cold PBS, liver contents were fixed in neutral buffered formalin. All animal experiments were approved by the Animal Care and Use Committee of the First Affiliated Hospital, College of Medicine, Zhejiang University (Reference number: 2019–1096).

### Histological analysis

Liver sections were fixed in neutral buffered formalin overnight and then embedded in paraffin blocks. Sections were cut and stained for Hematoxylin and Eosin (H & E). For determination of hepatic lipid accumulation, 8 μm frozen liver sections were sequentially stained with Oil Red O and hematoxylin (Jiancheng Biology, Nanjing, China). Cells grown in glass cover ships in 12-well plates were washed with PBS, and followed by staining with Oil Red O and hematoxylin. Sections were imaged at 200× magnification (Olympus, Tokyo, Japan).

### Cell culture and uric acid treatment

The mouse hepatocytes cell line (AML-12) and human hepatoblastoma cell line (HepG2) were obtained from the Chinese Academy of Science (Shanghai, China). AML-12 cells were grown in DMEM/F12 medium supplemented with 10% FBS, and 1% penicillin/streptomycin, ITS Liquid Media Supplement, and Dexamethasone (40 μg/ml). HepG2 cells were grown in DMEM medium supplemented with 10% FBS and 1% penicillin/streptomycin. Cells were incubated at 37 °C in 5% CO_2._ To established the uric acid-stimulated cell models, AML-12 and HepG2 cells were treated with different concentrations of uric acid (250, 500, and 750 μmol/L) for 48 h.

### miRNA array

Total RNA was isolated and the RNA integrity was assessed using Agilent Bioanalyzer 2100 (Agilent Technologies, Santa Clara, CA). The total RNA was transcribed to double strand cDNA, then synthesized into cRNA and labeled with Cyanine-3-CTP. The labeled cRNAs were firstly hybridized onto the microarray, which were scanned by the Agilent Scanner G2505C (Agilent Technologies). Array images were analyzed to get raw data by using feature extraction software (version 10.7.1.1, Agilent Technologies). Genespring (version 14.8, Agilent Technologies) were conducted for the basic analysis of the raw data. The raw data was normalized with the quantile algorithm. The threshold set for up- and down-regulated genes was a fold change ≥1.5.

### Cell transfection

Mmu/hsa-miR-149-5p inhibitor (100 nM), mmu/hsa-miR-149-5p (50 nM), and their corresponding negative controls were purchased from RiboBio (Guangzhou, China). The siRNA oligonucleotides and over-expression plasmids of mouse or human FGF21, and their corresponding negative controls were also obtained from RiboBio. The miRNAs, siRNAs and plasmids were transfected into hepatocytes by using Lipofectamine3000 (Invitrogen, Carlsbad, CA) according to the manufacturer’s instructions. After 24 h of transfection, the hepatocytes were exposed to uric acid for an additional 48 h.

### RNA isolation and real-time PCR

Total RNA was prepared from cells or frozen livers using RNA plus (Takara, Dalian, China). The 2.5 μg total RNA was reversely transcribed with a One Step PrimeScript™ RT-PCR kit (Takara). Real-time PCR analysis was carried out using the SYBR Premix-Ex Tag Kit (Takara) on an ABI prism 7500 sequence Detection System (Applied Biosystems, Foster City, CA). The primer sequences are listed in Table S1.

### Western blot analysis

Cells and liver tissues were homogenized by using RIPA buffer (Applygen Technologies Inc., Beijing, China) supplemented with protease and phosphatase inhibitor (Pierce Biotechnology, Rockford, IL). Equal amount of protein was subjected to 12% SDS-PAGE followed by transfer to PVDF membranes (Millipore, Inc., Darmstadt, Germany). Membranes were blocked with 5% non-fat dry milk in TBST, followed by incubation overnight with the following primary antibodies: anti-FGF21 (Abcam, ab17194, 1:1000), anti-β-actin (CST, 3700 s, 1:1000). Bolts were further incubated with HRP-conjugated secondary antibodies: goat anti-rabbit (Sigma, sc-2004) or goat anti-mouse (Sigma, sc-2005). Proteins were visualized with an ECL plus (Fudebio, Hangzhou, China).

### Triglyceride analysis

The intrahepatic and intracellular triglyceride contents were detected by using a commercial kit (Applygen Technologies Inc., Beijing, China) according the manufacturer’s instructions. The triglyceride values were normalized by total protein contents.

### Luciferase assay

To perform the luciferase reporter, plasmids containing either wild type or mutated 3’UTR sequence were generated (Hanyin, Shanghai, China). The plasmids were transfected into HEK293T cells using Lipofectamine 3000 (Invitrogen, Carlsbad, CA). The cells were also co-transfected with either the negative control or miR-149-5p mimic (50 nM). After 24 h of transfection, the cells were harvested according to the manufacturer’s instructions (Promega Dual Luciferase Assay Kit, Promega). Firefly luciferase values were normalized to those of Renilla luciferase.

### Statistical analyses

Statistical analyses were performed by using SPSS 18.0 for windows (SPSS, Chicago, IL). Statistical comparisons were made using *t*-test or ANOVA where appropriate. All data were expressed as mean ± standard division (SD), with a statistically significant difference defined as *P* < 0.05.

## Results

### Uric acid induces lipid accumulation in hepatocytes

**Fig. 1:**
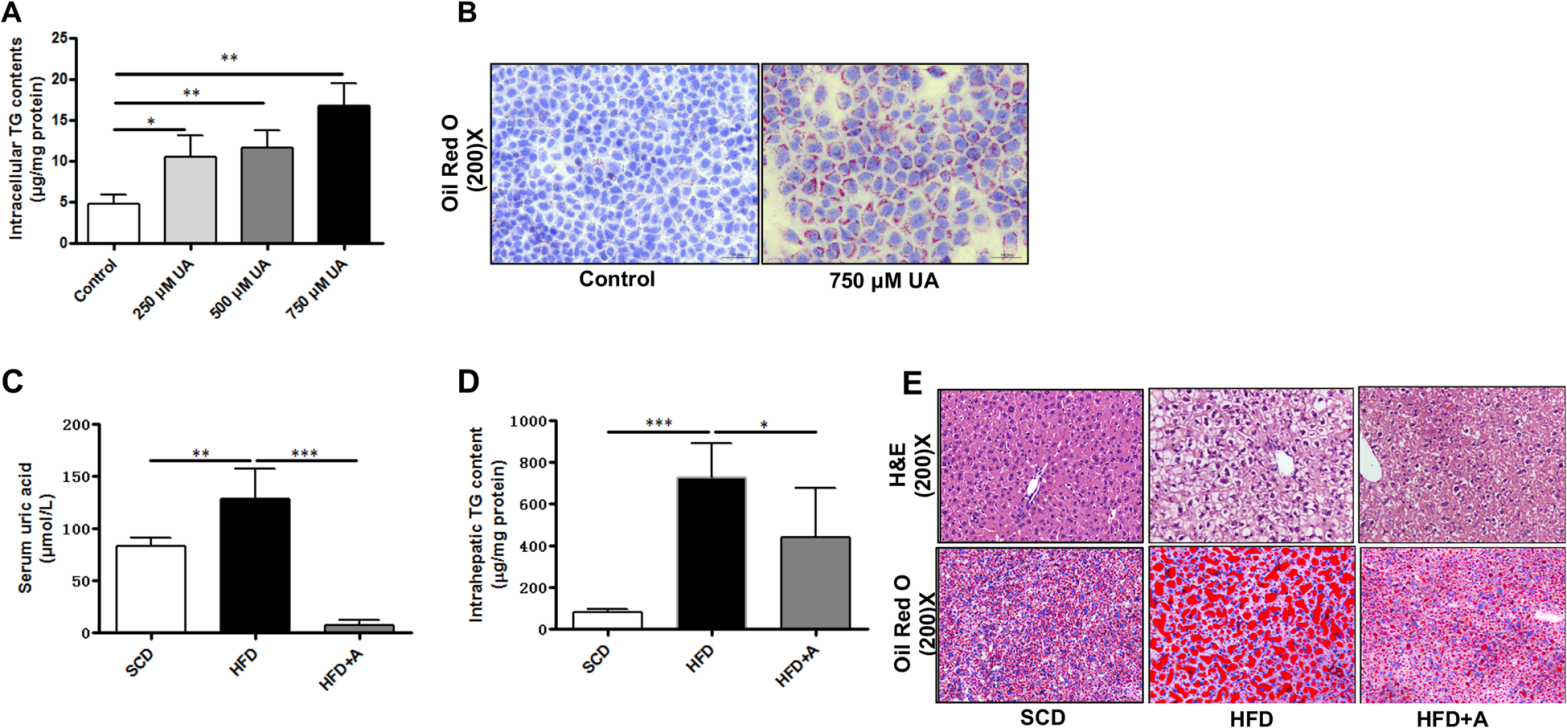
Uric acid induces hepatocytes lipid accumulation both in vitro and in vivo. **a** Intracellular triglyceride contents were significantly increased in AML-12 cells after stimulating with uric acid for 48 h; **b** Representative images of Oil Red O staining of AML-12 cells (200×). **c** Serum uric acid level was significantly higher in HFD-fed mice than SCD-fed mice, and the level was decreased by allopurinol treatment in HFD + A group. **d** Intrahepatic triglyceride contents of the mice fed by different diets. **f** Representative images of liver histology determined by H & E and Oil Red O staining (× 200). Data are presented as the mean ± SD of at least three independent replicates. * *P* < 0.05, ** *P* < 0.01, *** *P* < 0.001 of two-tailed student’s *t*-test or ANOVA

To investigate the regulatory role of uric acid on lipid accumulation in hepatocytes, we treated AML-12 cells with different concentrations of uric acid as previously reported [10]. We found that exposure to 250, 500, and 750 μmol/L uric acid for 48 h induced significant intracellular triglyceride accumulation in AML-12 cells (Fig. 1a). The increased intracellular lipid accumulation was confirmed by Oil Red O staining (Fig. 1b). We observed similar results in HepG2 cells treated with different concentrations of uric acid (Fig. S1). We also investigated the effect of uric acid on hepatic lipid accumulation in mice. Male C57BL/6 mice, 8 weeks of age, weighing 22–23 g, were randomly divided into three groups, consisting of 6 animals per group and fed with SCD, HFD, or HFD combined with allopurinol in drinking water (HFD + A), respectively. We found that the serum uric acid levels were significantly increased about 1.5-fold in HFD-fed mice, while the serum uric acid levels are reduced by about 90% in HFD + A mice than of HFD-mice (Fig. 1c). Consistent with the alterations of serum uric acid levels, we found that the intrahepatic triglyceride contents were reduced approximately by 40% in HFD + A fed mice than of HFD-fed mice (Fig. 1d). Liver sections with H & E and Oil Red O staining confirmed the reduction of hepatic lipid accumulation in HFD + A fed mice (Fig. 1e). These results suggested that uric acid directly induced hepatocytes lipid accumulation, and lowering serum uric acid by allopurinol significantly ameliorated HFD-induced hepatic steatosis in mice.

### Differentially expressed miRNA in HFD and HFD + A fed mice

**Fig. 2:**
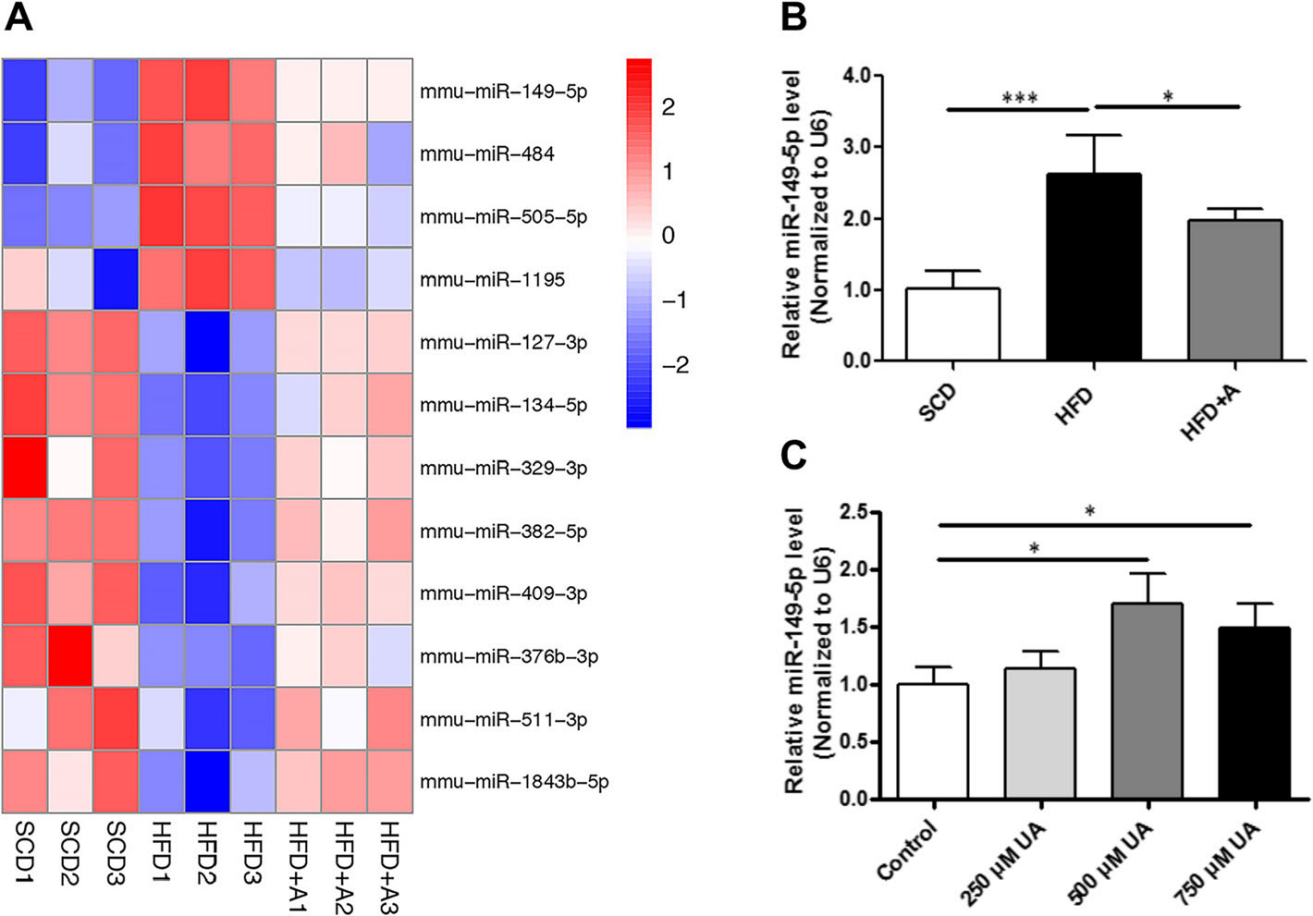
The differentially expressed miRNAs in livers of SCD, HFD and HFD + A fed mice. **a** Hierarchical clustering of the differentially expressed miRNAs in the livers of SCD, HFD and HFD + A fed mice. **b** Real-time PCR verification of miR-149-5p expression in the livers of SCD, HFD, and HFD + A fed mice. **c** Real-time PCR verification of miR-149-5p expression in AML-12 cells exposed to different concentrations of uric acid in AML-12 cells. Data are presented as the mean ± SD of at least three independent replicates. * *P* < 0.05, *** *P* < 0.001 of two-tailed student’s *t*-test

Microarrays were applied to explore the effect of uric acid on hepatic miRNA expressions in mice fed with SCD, HFD and HFD + A. We found that 47 miRNAs were differentially expressed at least 1.5-fold between the HFD and SCD groups, including 28 down-regulated and 19 up-regulated miRNAs. Furthermore, 26 miRNAs were differentially expressed at least 1.5-fold in HFD + A group compared with the HFD groups, including 5 down-regulated and 21 up-regulated miRNAs. We firstly identified that 12 miRNAs were both differentially expressed in HFD vs SCD, and HFD + A vs HFD (Fig. 2a, Table S2), indicating that those 12 miRNAs may both involved in the progress of high uric acid-induced hepatic steatosis and lowering uric acid-ameliorated hepatic steatosis.

Real-time PCR was performed to confirm the expression of differentially expressed miRNAs. Consistent with miRNA array analysis, we found that the expression of miR-149-5p was up-regulated about 2.5-fold in the livers of HFD-fed mice compared with SCD-fed mice, while the expression was down-regulated approximately by 20% in HFD + A mice compared with HFD-fed mice (Fig. 2b). We also found that miR-149-5p was significantly up-regulated about 1.5-fold in AML-12 cells exposed to 500 μmol/L and 750 μmol/L uric acid for 48 h (Fig. 2c). Similar results were observed in HepG2 cells (Fig. S2). These results indicated that the miR-149-5p may be associated with uric acid-induced hepatocytes lipid accumulation.

### Effect of miR-149-5p on lipid accumulation in hepatocytes

**Fig. 3:**
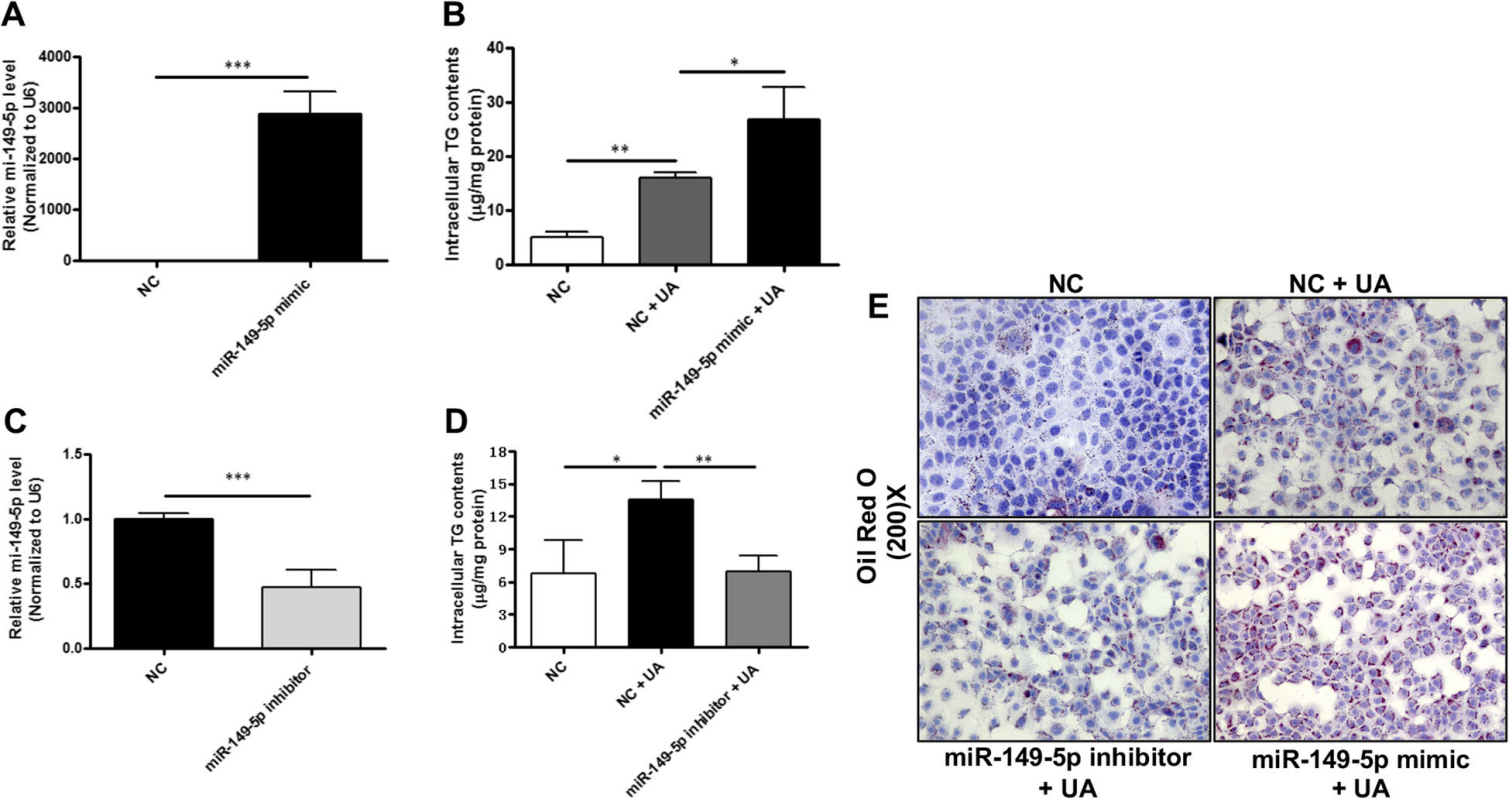
MiR-149-5p mediated the regulatory effects of uric acid on lipid accumulation in hepatocytes. **a** Real-time PCR confirmed miR-149-5p mimic increased miR-149-5p expression in AML-12 cells. **b** Overexpression of miR-149-5p aggregated uric acid-induced intracellular triglyceride accumulation in AML-12 cells. **c** Real-time PCR confirmed that miR-149-5p inhibitor decreased miR-149-5p expression in AML-12 cells. **d** Inhibition of miR-149-5p alleviated uric acid-induced intracellular triglyceride accumulation in AML-12 cells. **e** Oil Red O staining conformed the regulatory roles of miR-149-5p on uric acid-induced hepatocytes lipid accumulation (× 200). Data are presented as the mean ± SD of at least three independent replicates. * *P* < 0.05, ** *P* < 0.01, *** *P* < 0.001 of two-tailed student’s *t*-test

To verify whether miR-149-5p mediates the regulatory effects of uric acid on lipid accumulation in hepatocytes, loss- or gain-of-function of miR-149-5p on uric acid-stimulated hepatocytes was analyzed. miR-149-5p was overexpressed in AML-12 cells by transfection of miR-149-5p mimic (Fig. 3a). We found that overexpression of miR-149-5p significantly aggregated uric acid-induced intracellular triglyceride accumulation in AML-12 cells (Fig. 3b). On the other hand, inhibiting miR-149-5p expression by miR-149-5p inhibitor significantly ameliorated uric acid-induced triglyceride accumulation in AML-12 cells (Fig. 3c and d). Oil Red O staining of hepatocytes confirm the altered intracellular lipid accumulation (Fig. 3e). Similar results were observed in uric acid-stimulated HepG2 cells treated with miR-149-5p mimic or inhibitor (Fig. S3). These results suggested that miR-149-5p was involved in the regulatory effects of uric acid on lipid accumulation in hepatocytes.

### FGF21 is a target gene of miR-149-5p involved in uric acid-induced hepatocytes lipid accumulation

**Fig. 4:**
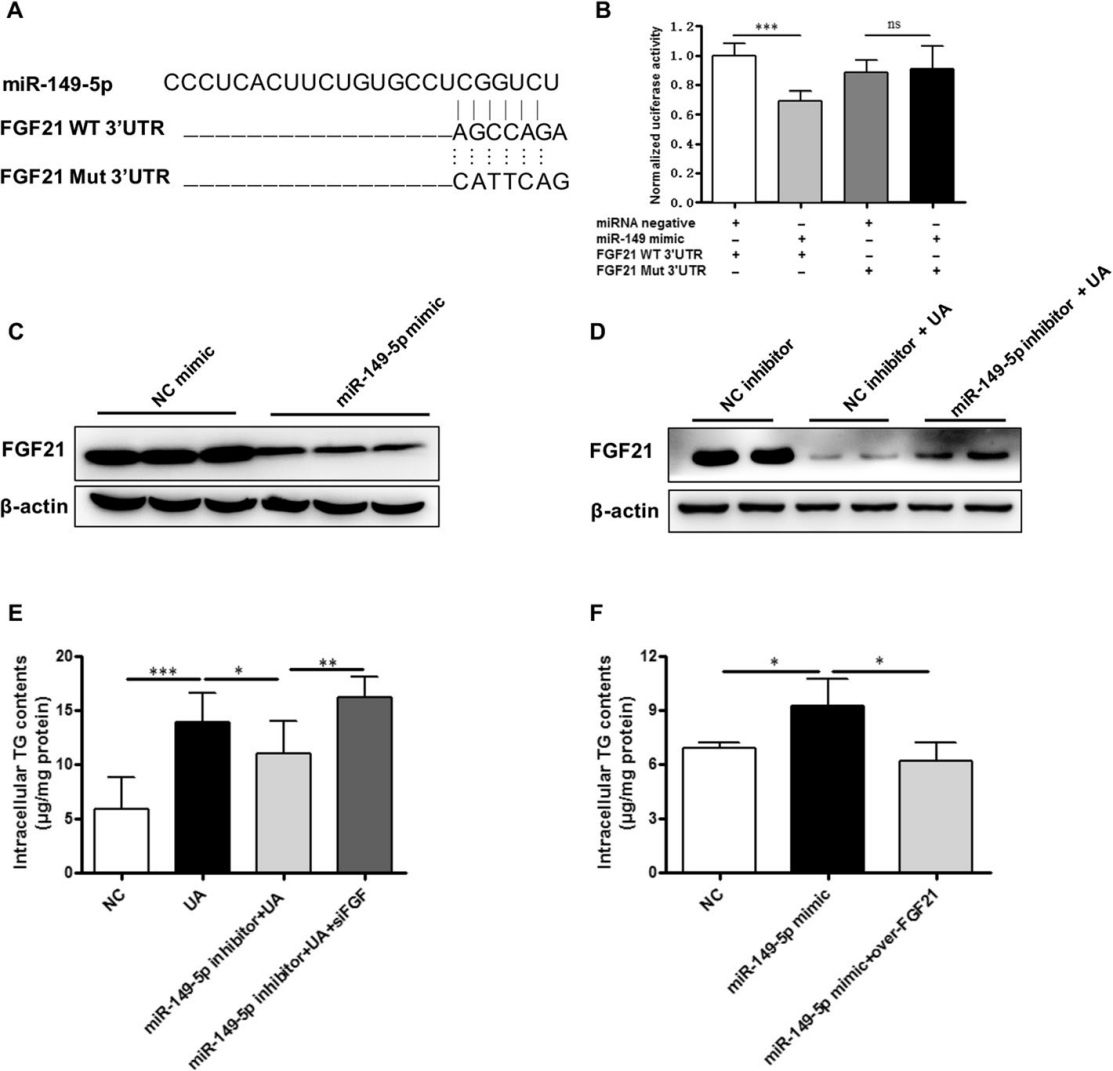
FGF21 is a downstream target of miR-149-5p that mediated uric acid-induced lipid accumulation in hepatocytes. **a** Diagram showing miR-149-5p binding sites predicted by miRwalk in the FGF21 3’UTR region. **b** Luciferase activity assays showed that miR-149-5p mimic significantly reduced the luciferase activity of wild type but not mutant 3’UTR of FGF21. **c** Western blot confirmed that miR-149-5p mimic significantly inhibited FGF21 expression. **d** Uric acid stimulation significantly down-regulated FGF21 expression, while miR-149-5p inhibitor restored the expression of FGF21 in uric acid-stimulated AML-12 cells. **e** Inhibiting FGF21 abolished the ameliorative effect of miR-149-5p inhibitor on uric acid-induced triglyceride accumulation in AML-12 cells. **f** Overexpression of FGF21 prevented the triglyceride accumulation induced by miR-149-5p mimic in AML-12 cells. Data are presented as the mean ± SD of at least three independent replicates. * *P* < 0.05, ** *P* < 0.01, *** *P* < 0.001 of two-tailed student’s *t*-test

By searching miRwalk prediction database, we found that FGF21 was predicted to be a target gene of miR-149-5p. FGF21 is a well-known protein that plays a key role in lipid metabolism [21, 22]. In order to determine whether FGF21 was a target gene of miR-149-5p, renilla luciferase reporter plasmids was constructed with the insertion of predicted miR-149-5p binding sites inside of human FGF21 (Fig. 4a). Co-transfection of miR-149-5p mimic led to a 30% reduction of the luciferase activity from the FGF21 WT 3’UTR in 293 T cells (Fig. 4b). However, mutation of predicted binding sites recovered the loss of luciferase activities mediated by excess miR-149-5p (Fig. 4b). Moreover, we also examine whether miR-149-5p regulated FGF21 endogenously. We found that transfecting with miR-149-5p mimic markedly suppressed the protein levels of FGF21 in AML-12 cells (Fig. 4c). Uric acid stimulation also suppressed the protein levels of FGF21 in AML-12 cells, whereas miR-149-5p inhibitor restored the expression of FGF21 in uric acid-stimulated hepatocytes (Fig. 4d). Similar results were observed in uric acid-stimulated HepG2 cells (Fig. S4a and b). These results suggested that miR-149-5p target regulated FGF21 through translation inhibition.

We further applied FGF21 siRNA and overexpression plasmids to determine the effect of FGF21 on lipid accumulation in hepatocytes. We found that silencing FGF21 abolished the ameliorative effect of miR-149-5p inhibitor on uric acid-induced triglyceride accumulation in hepatocytes (Fig. 4e). On the contrary, overexpression of FGF21 significantly prevented the triglyceride accumulation induced by miR-149-5p mimic in AML-12 cells (Fig. 4f). Similar results were observed in HepG2 cells treated with FGF21 siRNA and overexpression plasmids (Fig. S4c and d). These results suggested that FGF21 is the downstream target of miR-149-5p and mediates the regulatory effects of uric acid on lipid accumulation in hepatocytes.

## Discussion

In this study, we provide novel evidence that uric acid-induced hepatocytes lipid accumulation through miR-149-5p/FGF21 dependent mechanism. First, uric acid stimulation significantly up-regulated miR-149-5p expression in hepatocytes, while lowering uric acid by allopurinol significantly down-regulated hepatic miR-149-5p expression in HFD-fed mice. Second, loss- or gain-of-function studies found that miR-149-5p was responsible for pathogenic effect of uric acid on hepatocytes lipid accumulation. Third, we confirmed that FGF21 is a downstream target of miR-149-5p and FGF21 mediated the regulatory effects of uric acid on hepatocytes lipid accumulation.

Our previous studies have shown that SUA levels are significantly elevated in NAFLD patients, and that elevated SUA levels are associated with increased risk of incident NAFLD [9, 23]. Other large population-based cohort studies also found that high SUA levels are positively associated with risk of NAFLD [24, 25]. Histological studies have demonstrated that the SUA levels were significantly associated with histological disease severity of NAFLD [26–28]. Moreover, uric acid-lowering therapy by allopurinol significantly alleviated HFD-induced hepatic steatosis in an animal model of NAFLD [29]. These studies suggested that high uric acid may be a cause for the development of NAFLD, while its underlying molecular mechanism remains unclear.

MiRNA has been implicated as a crucial regulator in metabolic diseases by silencing genes at the post-transcriptional level. Growing evidence demonstrated that a large number of miRNAs, such as miR-122, miR-378 and miR-34a, are involved in the pathogenesis of NAFLD [30, 31]. However, whether miRNAs are involved in the uric acid-induced hepatocytes lipid accumulation is unclear. In this study, we aimed to explore the hepatic miRNA expression profiles of HFD-fed mice treated with or without allopurinol-therapy. In this study, we analyzed the miRNA profiles altered in response to HFD, and HFD combined with lowering uric acid therapy. We found that 47 miRNAs were differentially expressed between the HFD and SCD groups, including 19 up-regulated miRNAs and 28 down-regulated miRNAs. This finding is partially consistent with the previously observed in ob/ob mice [17, 32], MCD-fed mice [33], and NASH patients [34]. Our study confirmed that miRNAs are participated in the pathogenesis of NFALD.

We also found that 12 miRNAs were both differentially expressed in HFD vs SCD, and HFD + A vs HFD, suggesting potential involvement of those miRNAs in the progress of uric acid-induced hepatocyte lipid accumulation. Specifically, miR-149-5p was 5.31-fold up-regulated in HFD vs SCD, and 2.29-fold down- regulated in HFD + A vs HFD. Because miR-149-5p was the most obvious up-regulated miRNAs in response to HFD, we focused on this miRNA and explored its underlying mechanism on uric acid-induced hepatocytes lipid accumulation. MiR-149 was reported to be both a tumor suppressor and an onco-miRNA in various types of cancers, and has distinct functions including proliferation inhibition, apoptosis induction, cell-cycle regulation, and metastasis promotion separately [35–37]. A recent study reported that deficient of miR-149 increased mice whole-body energy expenditure, combined with enhanced thermogenesis of inguinal fat [38]. This finding suggested that miR-149 may play a potential role in the development of metabolic diseases. However, the role of miR-149-5p in NAFLD remains unclear. Here, we found that the expression of miR-149-5p was significantly elevated in response to uric acid stimulation in hepatocytes. Overexpression of miR-149-5p aggravated uric acid-induced hepatocytes lipid accumulation, whereas inhibition of miR-149-5p had the opposite effects. These results suggested that miR-149-5p mediated the regulatory of uric acid on hepatocytes lipid accumulation.

Furthermore, we also explored the down-stream mechanism of miR-149-5p on hepatocytes lipid accumulation. FGF21 is a member of the endocrine FGF family, mainly expressed and secreted in the liver and adipose tissue. FGF21 is an important regulator of lipid and glucose metabolism and plays a key role in the development in NAFLD [39, 40]. Some clinical studies reported that serum FGF21 level is increased in NAFLD patients and is considered to be a predictor of NAFLD [41]. However, recent studies reported different results that deficient of FGF21 induced hepatic steatosis [42], and overexpression of FGF21 was able to ameliorate liver fat accumulation [43]. In our study, we confirmed that FGF21 is a down-stream target of miR-149-5p in the process of uric acid-induced hepatocytes lipid accumulation. Our results suggest a new mechanism of uric acid-induced hepatocytes lipid accumulation.

There were several limitations in this study. First, the function and regulation of miRNA are complex, FGF21 may not be the only and the master target gene regulated by uric acid, further investigations are required to clarify the detailed downstream mechanisms of miR-149-5p on uric acid-induced hepatic steatosis. Second, although our in vitro cell line results clearly showed that uric acid regulates hepatocyte lipid accumulation through miR-149-5p/FGF21 dependent mechanism, primary hepatocytes experiments and in vivo experiments are needed to further confirm the regulatory mechanisms.

## Conclusions

In conclusion, our study provided evidence for the first time that uric acid induced hepatocytes lipid accumulation through miR-149-5p/FGF21 dependent mechanism.

## Supplementary information

**Additional file 1: Fig. S1.** Uric acid stimulation induced significant lipid accumulation in HepG2 cells. (a) Intracellular triglyceride contents in HepG2 cells exposed to different concentrations of uric acid for 48 h. (b) Representative image of Oil Red O staining of HepG2 cells (× 200). Data are presented as the mean ± SD of at least three independent replicates. * *P* < 0.05, ** *P* < 0.01 of two-tailed student’s *t*-test.

**Additional file 2: Fig. S2.** Real-time PCR verification of miR-149-5p expression in HepG2 cells exposed to different concentrations of uric acid for 48 h. Data are presented as the mean ± SD of at least three independent replicates. * *P* < 0.05 of two-tailed student’s *t*-test.

**Additional file 3: Fig. S3.** miR-149-5p mediated the regulatory effects of uric acid on intracellular lipid accumulation in HepG2 cells. (a) miR-149-5p mimic increased miR-149-5p expression levels in HepG2 cells. (b) miR-149-5p mimic enhanced uric acid-induced intracellular triglyceride accumulation in HepG2 cells. (c) miR-149-5p inhibitor decreased miR-149-5p expression levels in HepG2 cells. (d) miR-149-5p inhibitor ameliorated uric acid-induced intracellular triglyceride accumulation in HepG2 cells. (e) Oil Red O staining conformed the regulatory roles of miR-149-5p on uric acid-induced intracellular lipid accumulation in HepG2 cells (× 200). Data are presented as the mean ± SD of at least three independent replicates. * *P* < 0.05, ** *P* < 0.01 of two-tailed student’s *t*-test.

**Additional file 4: Fig. S4.** FGF21 is a target gene of miR-149-5p. (a) Western blot confirmed that miR-149-5p mimic significantly inhibited FGF21 expression in HepG2 cells. (b) Uric acid stimulation significantly down-regulated FGF21 expression, while miR-149-5p inhibitor restored the FGF21 expression in uric acid-stimulated HepG2 cells. (c) Silencing FGF21 abolished the ameliorative effect of miR-149-5p inhibitor on uric acid-induced intracellular triglyceride accumulation in HepG2 cells. (d) Overexpression of FGF21 decreased intracellular triglyceride contents induced by miR-149-5p mimic in HepG2 cells. Data are presented as the mean ± SD of at least three independent replicates. * *P* < 0.05 of two-tailed student’s *t*-test.

**Additional file 5: Table S1.** Primer sequences of genes analyzed by Real-time PCR.

**Additional file 6: Table S2.** Differential expressed miRNAs identified by microarray analysis of liver samples from SCD, HFD and HFD + A fed mice.

## Data Availability

The data that support the findings of this study are available from the corresponding author upon reasonable request.

## Abbreviations

DMEM: Dulbecco’s modified eagle medium
FBS: Fetal bovine serum
HFD + A: HFD combined with allopurinol therapy
HFD: High fat diet
miRNA: Microrna
NAFLD: Non-alcoholic fatty liver disease
NLRP3: NACHT, LRR and PYD domains-containing protein 3
PPARα: Peroxisome proliferator activated receptor alpha
SCD: Standard chow diet
SD: Standard division
siRNA: Small interfering RNA
UTR: Untranslated region
