# Supplementary figures and images for: Uric acid induced hepatocytes lipid accumulation through regulation of miR-149-5p/FGF21 axis
Source: BMC Gastroenterol. 2020 Feb 18;20:39. doi: 10.1186/s12876-020-01189-z (PMC7027271; doi:10.1186/s12876-020-01189-z)

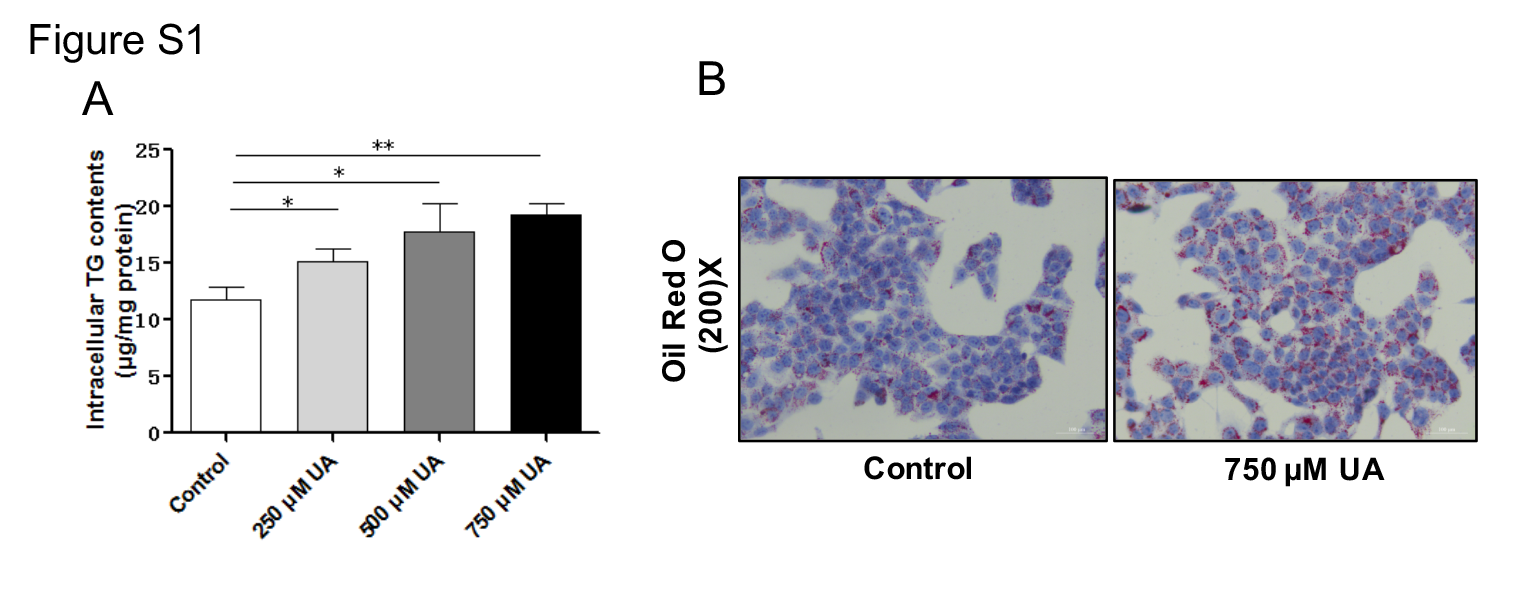

Supplement: Supplementary file 1 — Additional file 1: Fig. S1. Uric acid stimulation induced significant lipid accumulation in HepG2 cells. (a) Intracellular triglyceride contents in HepG2 cells exposed to different concentrations of uric acid for 48 h. (b) Representative image of Oil Red O staining of HepG2 cells (× 200). Data are presented as the mean ± SD of at least three independent replicates. * P < 0.05, ** P < 0.01 of two-tailed student’s t-test. [file 12876_2020_1189_MOESM1_ESM.tif]

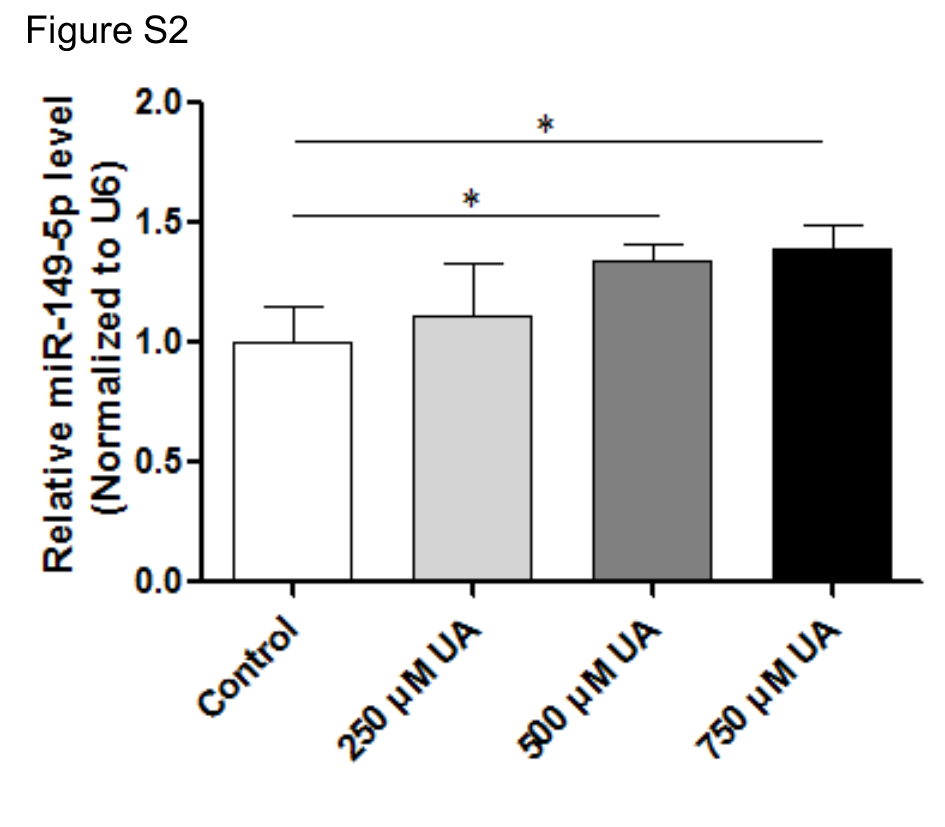

Supplement: Supplementary file 2 — Additional file 2: Fig. S2. Real-time PCR verification of miR-149-5p expression in HepG2 cells exposed to different concentrations of uric acid for 48 h. Data are presented as the mean ± SD of at least three independent replicates. * P < 0.05 of two-tailed student’s t-test. [file 12876_2020_1189_MOESM2_ESM.tif]

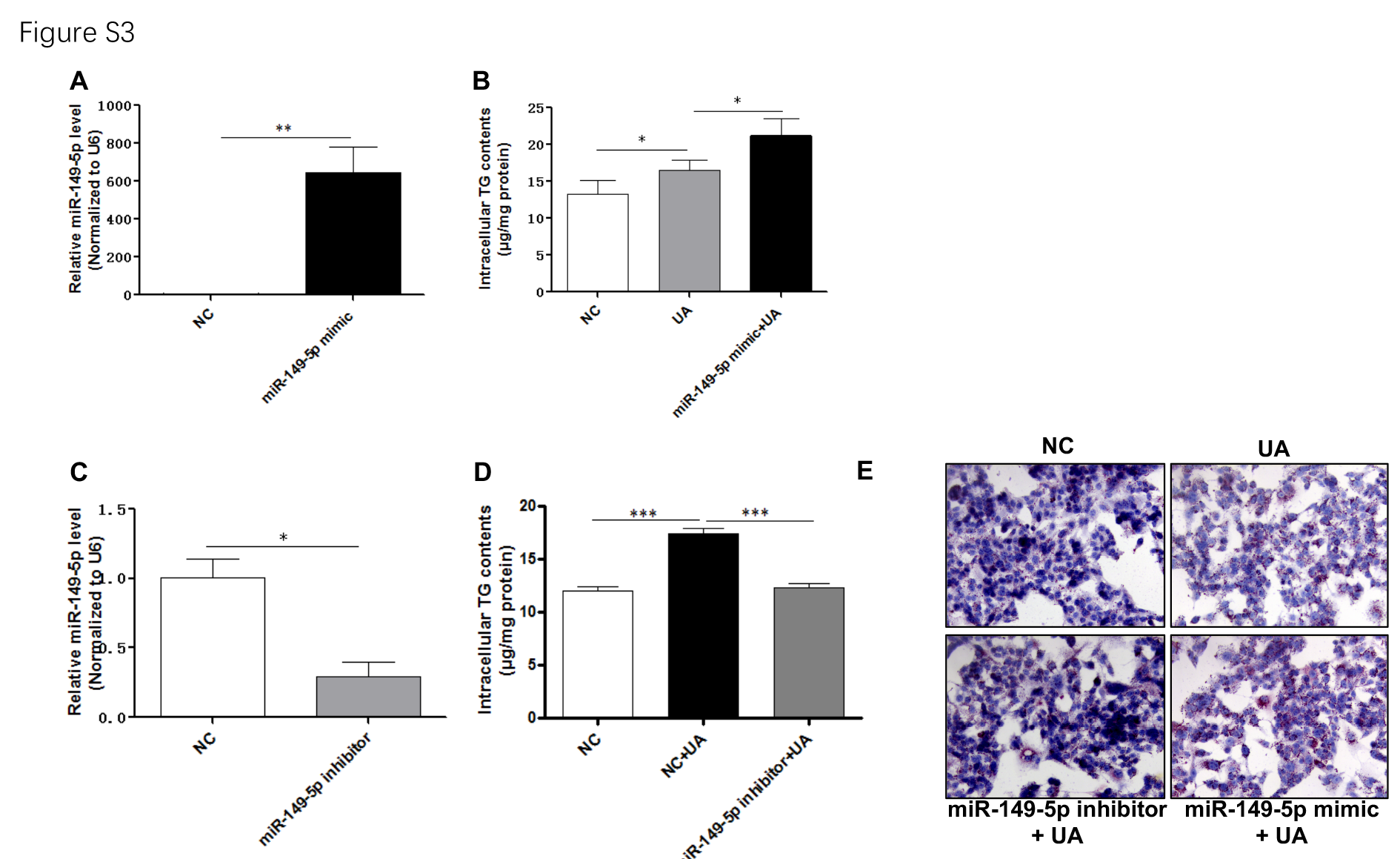

Supplement: Supplementary file 3 — Additional file 3: Fig. S3. miR-149-5p mediated the regulatory effects of uric acid on intracellular lipid accumulation in HepG2 cells. (a) miR-149-5p mimic increased miR-149-5p expression levels in HepG2 cells. (b) miR-149-5p mimic enhanced uric acid-induced intracellular triglyceride accumulation in HepG2 cells. (c) miR-149-5p inhibitor decreased miR-149-5p expression levels in HepG2 cells. (d) miR-149-5p inhibitor ameliorated uric acid-induced intracellular triglyceride accumulation in HepG2 cells. (e) Oil Red O staining conformed the regulatory roles of miR-149-5p on uric acid-induced intracellular lipid accumulation in HepG2 cells (× 200). Data are presented as the mean ± SD of at least three independent replicates. * P < 0.05, ** P < 0.01 of two-tailed student’s t-test. [file 12876_2020_1189_MOESM3_ESM.tif]

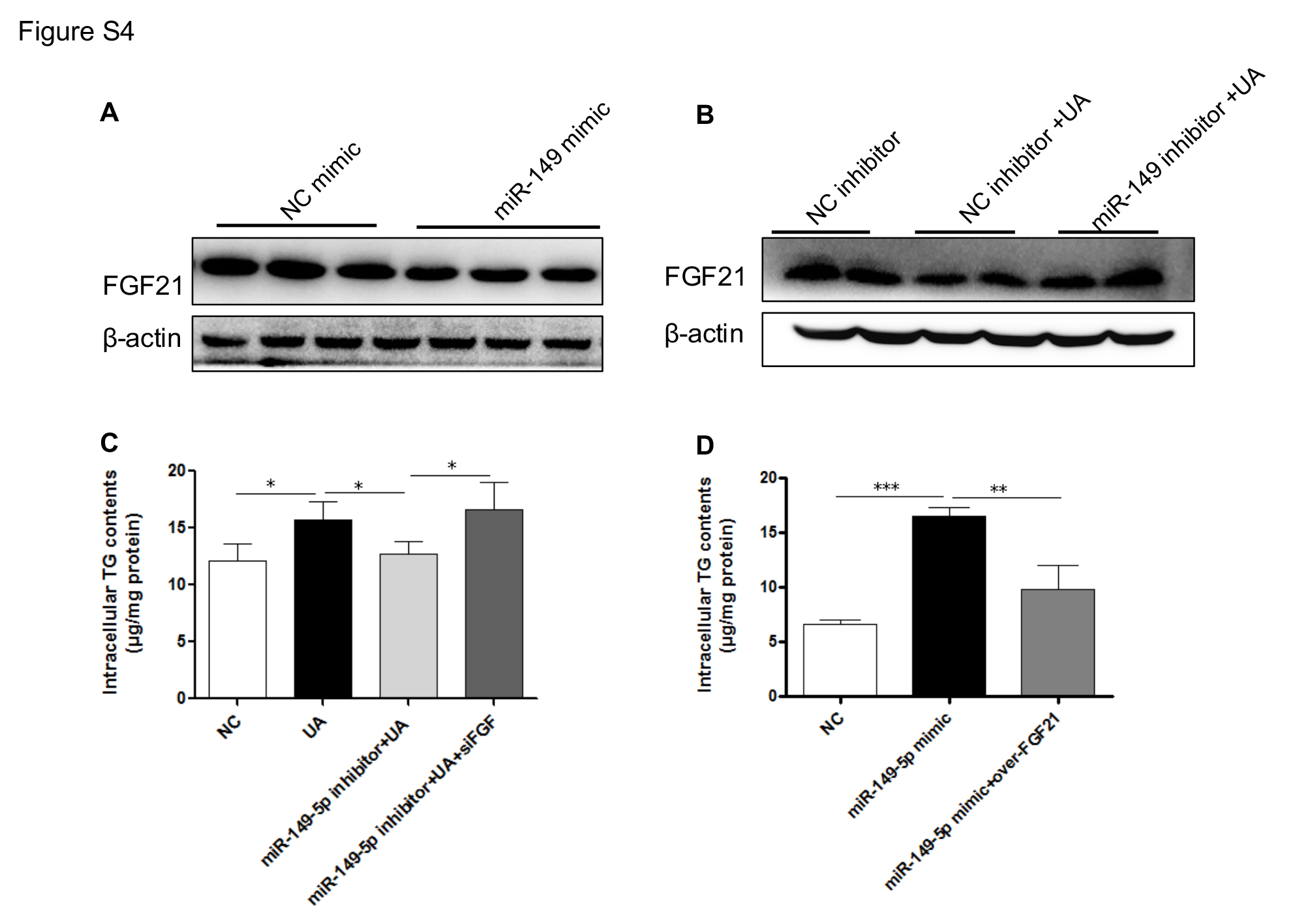

Supplement: Supplementary file 4 — Additional file 4: Fig. S4. FGF21 is a target gene of miR-149-5p. (a) Western blot confirmed that miR-149-5p mimic significantly inhibited FGF21 expression in HepG2 cells. (b) Uric acid stimulation significantly down-regulated FGF21 expression, while miR-149-5p inhibitor restored the FGF21 expression in uric acid-stimulated HepG2 cells. (c) Silencing FGF21 abolished the ameliorative effect of miR-149-5p inhibitor on uric acid-induced intracellular triglyceride accumulation in HepG2 cells. (d) Overexpression of FGF21 decreased intracellular triglyceride contents induced by miR-149-5p mimic in HepG2 cells. Data are presented as the mean ± SD of at least three independent replicates. * P < 0.05 of two-tailed student’s t-test. [file 12876_2020_1189_MOESM4_ESM.tif]
